# The paradox of immune checkpoint inhibition re-activating tuberculosis

**DOI:** 10.1183/13993003.02512-2021

**Published:** 2022-11-10

**Authors:** Mohamed Ahmed, Liku B. Tezera, Paul T. Elkington, Alasdair J. Leslie

**Affiliations:** 1 Africa Health Research Institute, Durban, South Africa; 2 College of Health Sciences, School of Laboratory Medicine and Medical Sciences, University of KwaZulu-Natal, Durban, South Africa; 3 NIHR Biomedical Research Centre, School of Clinical and Experimental Sciences, Faculty of Medicine, University of Southampton, Southampton, UK; 4 Dept of Infection and Immunity, University College London, London, UK; 5 Institute for Life Sciences, University of Southampton, Southampton, UK

## Abstract

By attenuating T-cell activation, immune checkpoints (ICs) limit optimal anti-tumour responses and IC inhibition (ICI) has emerged as a new therapy for a broad range of cancers. T-cell responses are indispensable to tuberculosis (TB) immunity in humans. However, boosting T-cell immunity in cancer patients by blocking the programmed cell death 1/programmed cell death ligand 1 (PD-1/PD-L1) axis can trigger re-activation of latent TB. This phenomenon appears to contradict the prevailing thought that enhancing T-cell immunity to *Mycobacterium tuberculosis* will improve immune control of this pathogen. In support of this anecdotal human data, several murine studies have shown that PD-1 deficiency leads to severe TB disease and rapid death. These observations warrant a serious reconsideration of what constitutes effective TB immunity and how ICs contribute to it. Through restraining T-cell responses, ICs are critical to preventing excessive tissue damage and maintaining a range of effector functions. Bolstering this notion, inhibitory receptors limit pathology in respiratory infections such as influenza, where loss of negative immune regulation resulted in progressive immunopathology. In this review, we analyse the mechanisms of ICs in general and their role in TB in particular. We conclude with a reflection on the emerging paradigm and avenues for future research.

## Abstract 2

**Immune checkpoint inhibition is employed as a host-directed therapy for cancer, but many reports have shown it can cause re-activation of latent TB. This observation warrants a reappraisal of protective TB immunity and drivers of re-activation.** https://bit.ly/3vi2xu0

## Introduction

A number of host-directed therapeutics (HDTs) have been licensed in recent years for the treatment of communicable and noncommunicable diseases through modulation of the host immune response. Perhaps the most successful of these has been the use of immune checkpoint inhibition (ICI) in the treatment of a number of cancers [1]. ICs consist of a family of receptors that are expressed on the surface of immune cells, particularly CD3 T-cells, and attenuate cellular activation through a variety of mechanisms [2]. These molecules are essential in promoting peripheral tolerance and preventing excessive immune responses that may result in immunopathology [3]. However, ICs can also act to hamper effective immunity, as in the case of certain anti-tumour responses, and their inhibition has proven to be a powerful therapeutic tool [4]. The commonly used inhibitors against a variety of tumour types consist of therapeutic monoclonal antibodies, such as pembrolizumab and ipilimumab, targeting the IC pathways of programmed cell death 1 (PD-1) and cytotoxic T-lymphocyte-associated protein 4 (CTLA-4), respectively. Blocking of these pathways re-invigorates anti-tumour T-cells, which are then able to effectively target the malignant cells and in many cases eradicate the tumour [5].

Despite generally favourable outcomes, a growing number of clinical reports have emerged of the re-activation of latent tuberculosis (TB) in patients undergoing ICI therapy to treat cancer. Multiple experimental studies in both human and animal systems have added support to these observations, and screening for latent TB is now seen as an important precaution for those patients undergoing ICI [6–8]. On the one hand, the development of progressive TB in the context of enhanced T-cell activity is somewhat counterintuitive, given the absolute requirement for T-cells in human TB immunity [9]. On the other hand, the fact that altering T-cell immunity through ICI can directly impact TB immunity, albeit negatively, does raise the intriguing possibility that the same pathways could be calibrated to produce the sort of positive effects that have been demonstrated in the field of cancer. It should also be noted from the outset, however, that ICs are expressed by a wide variety of cells and therefore re-activation following ICI may not be due entirely to the effect on T-cells. Here, we review the role of IC pathways in TB and the effects of inhibition, evaluate the possible mechanisms of TB disease progression caused by ICI, and assess the prospects of remodelled ICI to improve TB outcomes.

## Obstacles to protective TB immunity

Protective immunity against *Mycobacterium tuberculosis* in humans is a complex balance between host and pathogenic factors, the intricacy of which is not yet fully understood [10–13]. Indeed, despite years of research, the correlates of protective TB immunity remain largely unknown [14]. The role played by CD4 T-cells is, however, widely accepted to be critical, buttressed by extensive data from animal models and the fact that CD4 T-cell depletion in HIV infection severely weakens TB immunity [15–17]. Likewise, tumour necrosis factor (TNF)-α and the interleukin (IL)-12/interferon (IFN)-γ axis are thought to be essential components, as genetic deficiency in these signalling pathways has consistently been associated with increased risk of disease progression [18–21]. In addition, TNF-α blocking agents, used to treat chronic inflammatory diseases such as rheumatoid arthritis and Crohn's disease, led to numerous cases of TB re-activation [22, 23]. However, the fact that impaired immune signalling leads to disease susceptibility does not mean that an excess will be protective [24].

For most humans, natural immunity to TB appears to be highly effective and it is estimated that only 10% of infected individuals develop active TB disease in their lifetime [25]. In spite of this, a substantial portion of infected individuals may remain latently infected, suggesting that immunity is inadequate to prevent the establishment of persistent infection in the lung [26]. A small fraction of individuals, generally with some form of immunocompromise such as neonates and HIV patients, fail to prevent the early dissemination of infection and development of disease, referred to as primary TB or progressive primary TB [27]. This differs from post-primary TB affecting mostly adults, which occurs after the generation of systemic immunity and is marked by pulmonary cavities that facilitate transmission to a new host [28]. The immune failure associated with unresolved infection and/or progression to active TB in some individuals might be attributed to immunosuppressive mechanisms of *M. tuberculosis*, which first delay the initiation of adaptive immunity and subsequently evade the recognition of *M. tuberculosis*-infected cells by T-cells [29–33]. At the root of the problem, *M. tuberculosis* exhibits exceptional resistance to killing by macrophages, the first line of immune defence in the lung [34]. Inside macrophages, disruption of phagolysosome fusion and apoptosis by *M. tuberculosis* prevent efficient early bacterial clearance. In humans, adaptive immunity is detected 4–5 weeks after infection, providing ample time for prolonged bacterial replication [35]. Therefore, the rate at which T-helper (Th) type 1 (Th1) cells are activated in the lymph node and migrate to the lung has been suggested as a crucial factor in effective immune control [36, 37]. On the other hand, adoptive transfer of antigen-specific T-cells into naïve hosts before infection does not accelerate bacterial control and only confers protection 7 days post-infection [38]. In addition, there is no direct evidence linking the presence of Th1 T-cells in circulation or in the lung with protective TB immunity [39].

Many mechanisms have been put forward to explain the limits of T-cell immunity against *M. tuberculosis*. These include the influx of suppressive cell populations into the lung or that sustained antigen stimulation, resulting from a high bacterial burden, compromises T-cell functionality [40, 41]. Additionally, *M. tuberculosis* has been shown to subvert antigen presentation, in part by targeting major histocompatibility complex (MHC) II to limit CD4 T-cell activation [26, 32]. These, together with other possible mechanisms of T-cell impairment in TB, have been extensively reviewed [26, 31, 32]. In addition, it is now thought that there exists a diversity of infection both at an individual and population level, and that rigid classifications are inadequate to describe the features of the various manifestations of TB infection [42]. With these challenges in mind, examination of the regulation of ICs during TB infection might provide fresh insights into the host–pathogen interaction, persistent infection and development of active disease.

## Re-activation of TB following ICI

**FIGURE 1:**
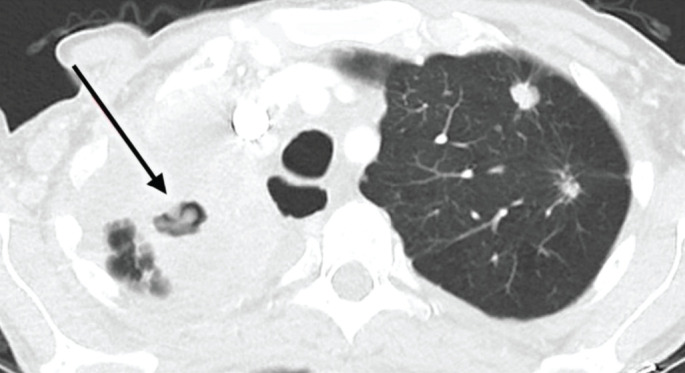
Computed tomography scan showing progressive tuberculosis disease (arrow) in the right lung of a cancer patient treated with nivolumab. Reproduced from [45] with permission.

The use of ICI to treat cancer represents a major conceptual breakthrough in the development of HDTs, despite being highly effective in only a minority of patients [43]. In addition, several groups have reported the development of active TB in patients as a side-effect of ICI (figure 1) [44, 45]. The first of these reports, by Lee *et al.* [46], described re-activation in a patient treated for Hodgkin lymphoma with the PD-1 inhibitor pembrolizumab. Next, Fujita *et al*. [47] reported on a case of acute TB after treatment with another PD-1 inhibitor, nivolumab, for stage IV lung cancer. Thereafter numerous reports describing similar observations as a consequence of ICI have been published including observations of the accompanying immune responses. An analysis of peripheral T-cell responses prior to and following anti-PD-1 treatment in a single cancer patient who developed TB re-activation is presented by Barber *et al.* [48]. Here, antigen-specific Th1 responses to *M. tuberculosis*, but not Th17 or CD8 T-cells responses, were detected 3 months after treatment with pembrolizumab. Despite the presence of *M. tuberculosis*-specific antibodies, consistent with prior infection, these Th1 responses were absent prior to ICI. Although only a study of one individual, the data presented by Barber *et al.* [48] may suggest that boosting Th1 T-cell function can lead to development of active, post-primary TB. However, as noted, PD-1 is expressed on a variety of immune cell subsets, which were not reported. Strikingly, all publications, bar one, describe the occurrence of TB disease due to PD-1/programmed cell death ligand 1 (PD-L1) inhibition. The exception involves a patient who initially received anti-CTLA-4 before continuing with anti-PD-1 and thus the involvement of CTLA-4 cannot be ruled out [49]. In addition to TB, Fujita *et al.* [50] recently reported on three cases of *Mycobacterium avium* re-activation in lung cancer patients undergoing anti-PD-1 therapy, suggesting that the re-activation mechanism may be conserved across different mycobacterial species. Recently, triple cancer therapy, involving both immunotherapy and chemotherapy concurrently, has been associated with several cases of TB re-activation [51, 52]. Analysis of the US Food and Drug Administration Adverse Events Reporting System between 2015 and 2020 revealed 72 cases of TB and 13 cases of atypical mycobacterial infection due to the use of PD-1/PD-L1 inhibitors [53]. Taken together with animal studies, these data identify anti-PD-1 therapy as deleterious to TB immunity, favouring disease re-activation. In contrast, there is no epidemiological data to suggest that CTLA-4 inhibition triggers TB re-activation [53].

## Mechanisms of ICs

**FIGURE 2:**
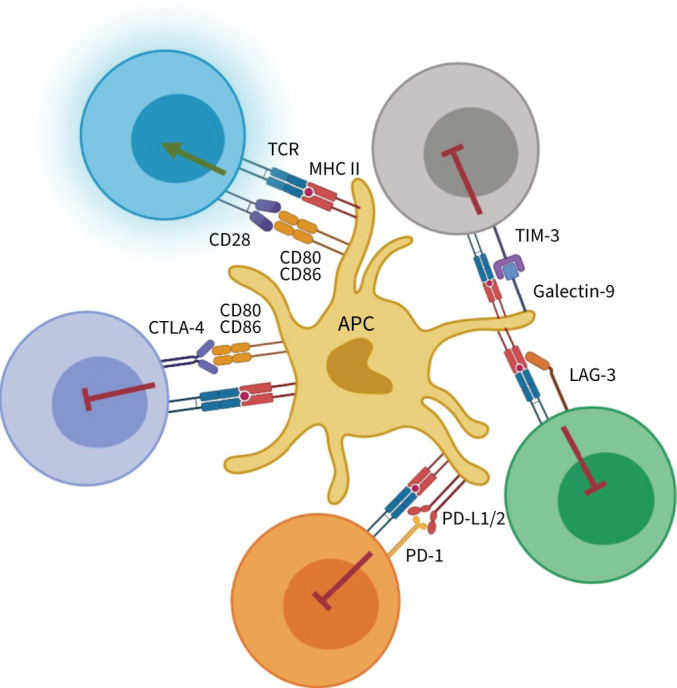
Immune checkpoint (IC) pathways that supress T-cell activation. The regulation of T-cell responses is dependent on the interaction with antigen-presenting cells (APCs). The expression of cognate antigen on major histocompatibility complex (MHC) molecules is recognised by the T-cell receptor (TCR). Secondly, CD80/CD86 on APCs provides “signal 2” to CD28 on T-cells. Together, these two signals induce T-cell activation (indicated by the green arrow). In contrast, ICs inhibit T-cell activation (indicated by the red “blocked” symbols) either as a host strategy to prevent excessive immune responses or as a function of pathology in order to supress immunity. CTLA-4: cytotoxic T-lymphocyte-associated protein 4; LAG-3: lymphocyte activation gene 3; PD-1: programmed cell death 1; PD-L1/2: programmed cell death ligand 1/2; TIM-3: T-cell immunoglobulin 3.

The immune system has evolved to defend against infections and then to rapidly return to tissue homeostasis [54, 55]. Disproportionate immune responses can inflict tissue damage and therefore close regulation of the immune response is required. IC molecules are now recognised as a key part of this process, by acting as brakes that avert excessive T-cell activation and subsequent immunopathology or autoimmunity [56]. Furthermore, restricting T-cell activity may preserve T-cell clones for future pathogen encounter, by preventing activation-induced cell death [3]. The relationship between IC expression and T-cell dysfunction is, however, complex. Generally speaking, the expression of any single IC molecule is considered a marker of T-cell activation rather than exhaustion [57, 58]. Indeed, naïve T-cells do not express IC molecules and their induction is directly correlated with T-cell receptor (TCR) signal strength [59–61]. In addition, tissue-resident memory T-cells, which are highly functional and critical to immunity at barrier sites, often express high levels of PD-1 [62, 63]. Exhaustion, on the other hand, is generally defined as defective effector function and is often linked to the sustained expression of IC molecules, and coexpression of several IC molecules is indicative of the severity of impairment [3, 64]. Moreover, ample evidence suggests the consequences of ICI on T-cell activity are not generic and are dependent on the specific pathways that are inhibited [3]. In other words, the blockade of certain IC pathways, or combination of pathways, may lead to divergent patterns of T-cell expansion and activity. Here, we present a brief overview of prominent IC molecules and the molecular and cellular mechanisms that govern their function (figure 2).

### PD-1

PD-1 plays a major role in the maintenance of central and peripheral tolerance, and in constraining T-cell responses [65]. PD-1 exerts its function by limiting signalling through both the TCR and the co-stimulatory molecule CD28, which provides the “second signal” required for T-cell activation through binding of its ligands CD80 and CD86 on the antigen-presenting cell (APC). Engagement of PD-1 by its ligands PD-L1 and PD-L2 results in activation of tyrosine phosphatase SHP2, which in turn inhibits signalling through the TCR and CD28. It has been proposed that the balance of activation of co-stimulatory molecules and inhibitory receptors functions as a rheostat to fine-tune T-cell responses [66]. In this manner the threshold of antigen responses of T-cells is regulated [67]. Expression of PD-1 ligands by nonhaematopoietic cells as well as haematopoietic cells, induced by inflammatory cytokines, helps to maintain homeostatic control and prevents tissue damage [68]. However, tumours upregulate PD-L1/2 in response to T-cell-derived IFN-γ in order to escape immunosurveillance and maintain a general anti-inflammatory milieu; a phenomenon known as “adaptive immune resistance” [69]. Thus, responsiveness to ICI therapy is correlated to the presence of pre-existing anti-tumour CD8 T-cells that express PD-1 and are thus shackled by PD-L1/2 expression on tumour cells [70, 71]. Consistent with this mechanism, anti-PD-1 treatment failed in patients whose tumours exhibit genetic defects in the IFN-γ pathway [72, 73]. Longitudinal examination of peripheral blood from stage IV melanoma patients identified PD-1^+^ CD8 T-cells as the main targets of PD-1 inhibition, which causes a marked expansion of an IFN-γ-producing CXCR5^+^PD-1^+^ subset [74]. Consistent with this, PD-1 blockade in chronically lymphocytic choriomeningitis virus (LCMV)-infected mice resulted in the expansion of CXCR5^+^PD-1^+^ CD8 T-cells [75]. This was further confirmed in human and murine tissues comparing CTLA-4 *versus* PD-1 inhibition [76].

Blockade of the PD-1/PD-L1 axis in both humans and animal models has been shown to improve immune control of infections such as malaria, hepatitis B and HIV [77]. In chronically LCMV-infected mice, PD-1/PD-L1 blockade, but not CTLA-4 blockade, significantly reduced viral load due to re-invigoration of exhausted CD8 T-cells [78]. In the same study, however, PD-L1 knockout (KO) mice were highly susceptible to LCMV infection, dying rapidly of immunopathology, highlighting the part played by the PD-1 axis in limiting tissue damage. Interestingly, genetic loss of PD-1 leads to the accumulation of terminally differentiated effector CD8 T-cells in LCMV-infected mice [79]. This finding demonstrates a probable role for PD-1 in protecting T-cell populations from exhaustion. Critically, this study shows that exhaustion can occur in the absence of PD-1, demonstrating that the molecule itself does not define an exhausted state. In addition, despite expressing high levels of PD-1, more terminally differentiated CD8 T-cells are less responsive to PD-L1 blockade, suggesting a threshold of exhaustion beyond which T-cell function cannot be restored [57]. It is still debated as to what precisely distinguishes exhausted and terminally differentiated T-cells, and discussion is made more complex by the fact that the terminology used can vary [80]. Therefore, the role of PD-1 in immune regulation is highly nuanced and the impact of inhibition appears to be very context dependent.

### CTLA-4

CTLA-4 also dampens T-cell activation by competing with CD28 for its ligands CD80 and CD86, expressed on APCs [81]. The structural similarity to CD28 and a stronger binding affinity to CD80 and CD86 allows CTLA-4 to outcompete CD28 for these ligands and so curtails T-cell activation [81, 82]. Interestingly, recent data suggest that PD-1-induced SHP2 mainly targets CD28, indicating a functional overlap between CTLA-4 and PD-1 [3]. Primarily, CTLA-4 regulates early T-cell priming in the lymphoid organs and controls activation in peripheral tissues. Genetic KO or antibody-meditated inhibition of CTLA-4 in mice causes aberrant expansion of several sets of effector CD4 T-cells, suggesting a key role for CTLA-4 in regulating T-cell expansion and differentiation [83]. CTLA-4 is critical for the function of regulatory T-cells (Tregs) and CTLA-4 blockade can impair this activity [84]. In Tregs, CTLA-4 acts both through competition with effector T-cells for the co-stimulatory ligands and by limiting the availability of these molecules by depleting them from the cell surface *via* transendocytosis [85, 86]. Indeed, CTLA-4 expression on Tregs is required for the maintenance of tolerance, as severe immune dysregulation is associated with Treg impairment in humans with CTLA-A deficiency [87–91]. Deletion of CTLA-4 in mice led to the development of severe autoimmune disease and lymphoproliferative disorder [92–94]. This is consistent with the role of CTLA-4 in eliminating autoreactive T-cells during naïve T-cell activation in the lymph nodes [95]. In contrast, PD-1 and T-cell immunoglobulin 3 (TIM-3) control T-cell activation at later stages, which might explain the lack of autoimmunity observed in mice lacking these other inhibitory receptors. CTLA-4 depletion in mice enhances antitumoral activity of CD8 T-cells and suppression of Tregs within the tumour micro-environment [96–99]. In both humans and mice, anti-CTLA-4 therapy resulted in an increase in ICOS^+^T-bet^+^ Th1-like CD4 effector T-cells as well as phenotypically exhausted CD8 T-cells [76, 100, 101]. Contradictory data exist, however, with respect to the impact of CTLA-4 blockade on infection control. CTLA-4 inhibition did not enhance resistance to *Toxoplasma gondii* while worsening murine malaria infection [102, 103]. In contrast, CTLA-4 inhibition accelerated clearance of *Listeria monocytogenes* in mice and enhanced HIV antibody induction in monkeys [104, 105]. Although anti-CTLA-4 and anti-PD-1 lead to the expansion of CD8 T-cells, anti-CTLA-4 alone appears to expand the CD4 T-cells compartment, underscoring the contrasting patterns of T-cell expansion seen in CTLA-4 *versus* PD-1 blockade [76].

### LAG-3 and TIM-3

Beyond PD-1 and CTLA-4, other molecules have emerged as potential targets for ICI, including lymphocyte activation gene 3 (LAG-3) and TIM-3 [106]. LAG-3 structurally resembles CD4 and binds MHC II molecules with higher affinity than CD4, likely transmitting inhibitory signals *via* its cytoplasmic domain [107, 108]. In addition, T-cell homeostasis is negatively regulated by LAG-3 *via* Treg-dependent mechanisms [109]. In both infection and cancer, coexpression of LAG-3 and PD-1 negatively regulates T-cell responses, which could be remedied by combined blockade [110–113]. TIM-3 engages its ligand, galectin-9, to suppress T-cell function by selectively inducing cell death of IFN-γ-producing Th1 cells [114]. Other TIM-3 ligands include the phospholipid PtdSer expressed on apoptotic cells, the alarmin high mobility group box 1 (HMGB1) that binds to DNA released from dying cells and the glycolipid CEA cell adhesion molecule 1 (CEACAM1) known to be highly expressed on tumour cells [115]. As is the case for LAG-3, CD8 T-cell activation is regulated by the coexpression of PD-1 and TIM-3, and functionality can be restored by dual blockade [116]. TIM-3 also functions as an inhibitory receptor on innate cells such as natural killer (NK) cells, macrophages and dendritic cells (DCs). Engagement of TIM-3 on NK cells significantly reduced cytotoxic capacity [117]. In macrophages, overexpression of TIM-3 impaired Toll-like receptor-mediated cytokine production, while blockade of TIM-3 enhanced macrophage activation and led to severe sepsis [118]. In accordance, downregulation of TIM-3 in peripheral blood mononuclear cells (PBMCs) from patients correlated with severity of sepsis, pointing to a protective role of TIM-3 in restraining excessive inflammation [118]. Anti-TIM-3 treatment improved anti-tumoural responses by promoting production of CXCL9 by CD103^+^ DCs following contact with tumour cell debris [119]. The effect of TIM-3 inhibition may vary depending on cell type as blockade is not cell selective.

## Modulation of ICs in TB

### Expression of PD-1/PD-L1 is upregulated in active TB

Expression profiles of ICs in TB patients have been characterised extensively over the years, although studies have primarily focused on the PD-1/PD-L1 axis. PD-1 expression has been consistently shown to be increased on circulating T-cells in patients with active TB disease compared with healthy controls [120–123]. Interestingly, the expression of PD-1 and its ligands (PD-L1 and PD-L2) is also markedly increased on monocytes during active infection [121, 124, 125]. Furthermore, several studies have shown PD-1 expression directly correlates with bacterial load and the magnitude of IFN-γ responses, suggesting antigen levels may be a driving factor [126, 127]. Indeed, PD-1 expression was increased on CD4 T-cells producing Th1 cytokines from smear-positive TB patients compared with smear-negative patients and latently infected subjects [127]. Here, stimulation with *M. tuberculosis* antigens induced PD-1 expression on *M. tuberculosis*-specific CD4 T-cells, indicating that its expression may be directly upregulated by the presence of antigen. These observations have recently been extended to TB infected lung tissue, where PD-1 expression was highest in T-cells expressing the markers of tissue residency, CD103 and/or CD69 [8]. However, while PD-L1 expression is widespread, immunohistochemical staining found PD-1 expression to be absent in caseating granulomas, potentially indicating a role for PD-1 in limiting immunopathology and granuloma progression [8, 49].

An early study comparing CTLA-4 expression between HIV-negative TB patients and healthy controls previously exposed to *M. tuberculosis* found CTLA-4 expression to be significantly reduced in TB patients compared with controls [128]. In the same patient group, CTLA-4 expression was significantly upregulated in response to IL-12 stimulation or IL-10 neutralisation. Another report failed to detect CTLA-4 expression on unstimulated PBMCs from active TB patients or patients who completed treatment [129]. However, both CTLA-4 and IFN-γ expression increased after bacille Calmette–Guérin (BCG) stimulation, and this was more pronounced in patients at treatment end compared with newly diagnosed cases. More recently, examination of *M. tuberculosis*-specific CD4 T-cells revealed significantly elevated expression of both CTLA-4 and PD-1 on these cells [130]. An increase in CTLA-4-expressing Tregs has recently been described in subjects with active pulmonary TB, which reduced following treatment [131].

A similar pattern emerges from the limited studies performed for the other IC molecules. LAG-3 expression was upregulated in the lungs of macaques with active disease, but not those with latent infection, and is detected in human lung granuloma-associated T-cells [132]. However, it was not detected on T-cells in the blood, which does raise the issue that the expression of IC molecules may differ between the blood compartment, where they are often measured in humans, and the site of disease. TIM-3 expression was substantially higher in both CD4 and CD8 TB-specific T-cells in the blood of patients with active disease compared with healthy controls [133] and was associated with disease severity [134], and in one study observed in conjunction with PD-1 [122]. Taken together, these data support a role of ICs in regulating the immune response to TB in humans, but the net effect on host immunity is unclear.

### IC expression is downregulated in response to TB therapy

The identification of biomarkers to determine TB treatment efficacy has garnered great interest in recent years [135, 136]. Several studies have demonstrated that PD-1, CTLA-4 and TIM-3 expression in the peripheral blood decreases significantly following TB treatment. In a TB-HIV cohort, concurrent antiretroviral therapy and TB therapy markedly reduced PD-1 and CTLA-4 expression in antigen-specific CD4 T-cells [130]. This was further substantiated by data showing PD-1 expression was only reduced in IFN-γ-expressing *M. tuberculosis*-specific CD4 T-cells after treatment, not in CD8 T-cells [127]. The decline of PD-1 expression during treatment was inversely related to the ratio of IFN-γ to IL-4, potentially indicating a restoration of protective immune properties [125]. Another study showed that effector T-cells exhibited the greatest decrease in PD-1 expression after treatment, with no differences observed in Tregs [120]. Importantly, treatment also induces downregulation of the PD-1 axis in innate cells, as significant downregulation of PD-L1 and PD-L2 in macrophages is associated with successful treatment [121]. On NK cells, PD-1 expression was decreased 2 months after treatment initiation [137]. Finally, expression of PD-1 and TIM-3 in CD8 T-cells was downregulated in *M. tuberculosis*-infected mice after receiving treatment [138]. Although not comprehensively proven, the preferential decrease of IC expression on antigen-specific cells suggests the elimination of antigen stimulation as a likely mechanism, at least for CD4 T-cells.

### Inhibition of ICs enhances effector functions

**FIGURE 3:**
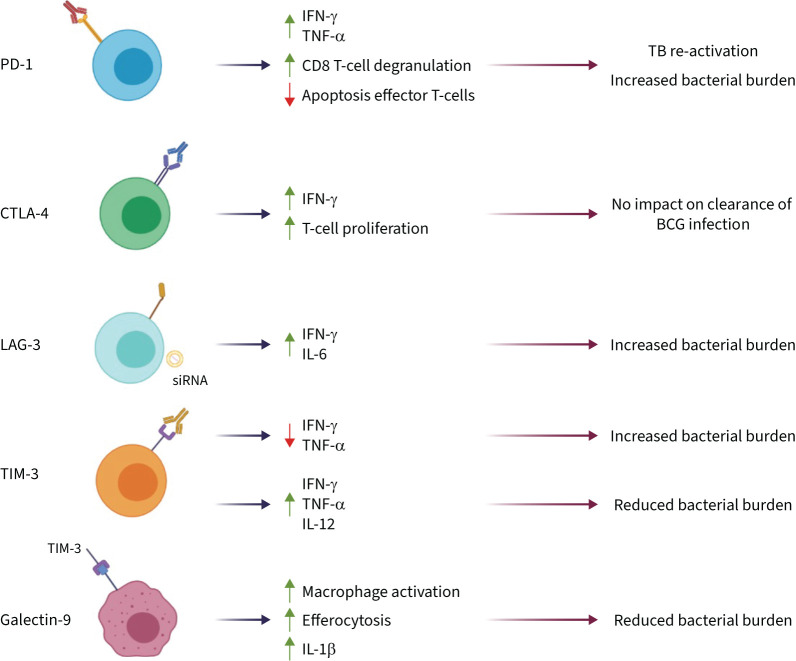
Effect of immune checkpoint inhibition (ICI) in tuberculosis (TB). Inhibition of IC molecules and downstream effects on TB pathology. Blockade by antibodies of programmed cell death 1 (PD-1), cytotoxic T-lymphocyte-associated protein 4 (CTLA-4) and T-cell immunoglobulin 3 (TIM-3). Lymphocyte activation gene 3 (LAG-3) is inhibited by small interfering RNA (siRNA). Galectin-9 is shown engaging its ligand TIM-3. IFN: interferon; TNF: tumour necrosis factor; IL: interleukin; BCG: bacille Calmette–Guérin.

Considering the inhibitory properties of IC molecules, it has been hypothesised that their inhibition could enhance effector functions in TB (figure 3). In a mouse model, CTLA-4 blockade resulted in increased lymphocyte numbers in the lymph node and antigen-induced IFN-γ secretion *in vitro* [139]. However, it did not affect clearance of BCG nor granuloma formation. *In vitro* blockade of CTLA-4 on expanded Tregs from subjects with active TB disease was recently shown to enhance IFN-γ production and the proliferation of *M. tuberculosis*-specific T-cells, and improve macrophage killing of *M. tuberculosis* [131]. Generally, *in vitro* inhibition of the PD-1/PD-L1 axis suggested possible improvements of both innate and adaptive cytokine production and killing potential. Inhibition of PD-1 and its ligands, for example, enhanced degranulation and IFN-γ production of CD8 T-cells and NK cells from TB patients [126, 140]. This effect could be further augmented in CD8 T-cells by simultaneous co-stimulation [126]. Two recent reports confirmed these findings in samples collected from TB pleurisy patients [125, 141]. Blockade of the PD-1 pathway increased the frequency of IFN-γ-producing T-cells as well as CD8 T-cell degranulation [141]. Specifically, PD-L1 inhibition enhanced CD8 T-cell cytotoxicity against pro-inflammatory macrophages compared with anti-inflammatory macrophages [124]. Inhibition of PD-1 prevented apoptosis of *M. tuberculosis*-specific IFN-γ-producing T-cells taken from TB patients [125]. Thus, while PD-1 expression may preserve T-cell function overall, inhibition of PD-1 may restore effector functions in exhausted populations (due to persistent antigen stimulation) present in TB patients.

Silencing of LAG-3 in lung-derived CD4 T-cells taken from macaques significantly reduced *M. tuberculosis* burden in co-cultured macrophages, while also promoting IFN-γ and IL-6 production [132]. Several studies have also investigated the modulation of TIM-3 to enhance TB immunity, with somewhat conflicting findings. For instance, TIM-3 blockade or ablation in mice enhanced T-cell function and moderately reduced bacterial burden, whereas stimulation of the TIM-3/galectin-9 axis promoted macrophage activation and also restricted bacterial replication mediated by IL-1β secretion [142–145]. In contrast to these findings, a separate study reported that TIM-3^+^ T-cells from TB patients more potently controlled *M. tuberculosis* growth in macrophages compared with TIM-3^−^ T-cells [133, 146]. Here, knockdown of the TIM-3 pathway using silencing RNA was found to impair IFN-γ production while stimulation further enhanced it. Although more work is needed to resolve some of these contrasting observations, together they highlight the differences between IC pathways and suggest potential differing roles in regulating the host–pathogen interaction.

### Loss of PD-1/PD-L1 signalling exacerbates disease

The lung is extremely sensitive to unchecked inflammation and inhibitory signals are imperative to minimise tissue damage [147]. Loss of the PD-1 axis consistently leads to worsening of TB disease. PD-1-deficient mice are highly susceptible to *M. tuberculosis* infection and died rapidly compared with wild-type mice [143, 148]. Severe necrotic pneumonia developed in the lungs of these PD-1 KO mice which is characterised by massive neutrophil infiltration and higher levels of IL-6, IL-17 and IFN-γ [148]. In a separate study, *M. tuberculosis* infection in PD-1-deficient mice was associated with decreased autophagy in macrophages, impaired proliferation of antigen-specific lymphocytes and increased Treg activity [149]. Subsequently, using a combination of adoptive transfer and depletion, it was shown that the reduction in survival was found to be mediated by PD-1 deficiency in CD4 T-cells with a minor role for CD8 T-cells, since depletion of CD4 T-cells rescued PD-1 KO mice from early mortality and severe lung pathology [6]. Likewise, PD-1 inhibition in macaques aggravates TB disease with larger sized granulomas in the lung [150]. However, in this system, it was accompanied specifically by increased frequencies of *M. tuberculosis*-specific CD8 T-cells that exhibited increased production of IFN-γ and IL-2 in the granulomas of anti-PD-1-treated animals. *M. tuberculosis*-specific CD4 T-cells appeared unaffected and the authors concluded that PD-1 blockade exacerbated disease primarily through CD8 T-cells. In contrast to *M. tuberculosis*, PD-1-deficient mice controlled BCG infection more effectively than wild-type mice [151]. This suggests that in the face of an attenuated strain, PD-1 expression may restrict clearance, but with a virulent strain like *M. tuberculosis* it is necessary to moderate inflammation. However, as noted earlier, anti-PD-1 therapy in humans is associated with an increased risk of atypical mycobacterial infections, generally considered to be less virulent than *M. tuberculosis* [152]. Along with apparent differences between the mechanism of action of PD-1 blockade in monkey and mouse models, these findings caution the direct translation of findings across the different models and human TB.

Augmentation of IFN-γ failed to confer protection *in vivo* or *in vitro* and evidence suggests IFN-γ may impair protective responses, all of which seems at odds with the concept of IFN-γ being indispensable to TB immunity [153–155]. Consistent with this, overexpression of IFN-γ in CD4 T-cells has been shown to accelerate death in mice, a fate that could be prevented by PD-1 expression [7]. Using an *in vitro* granuloma model, PD-1 blockade significantly increased TNF-α production in *M. tuberculosis*-infected human PBMCs [8]. This resulted in increased bacterial growth, which could be mitigated by TNF-α neutralisation. Potentially excessive TNF-α might be driving loss of macrophage function, as TNF-α was shown to induce necrosis in *M. tuberculosis*-infected macrophages mediated by production of reactive oxygen species [156, 157]. However, the source of TNF-α in these experiments was not established. Recently, PD-1 deficiency associated with TB disease in a child was linked to lower IFN-γ responses [158]. In addition, there may be other detrimental consequences of PD-1 deficiency, such as the requirement of PD-1 signalling for expansion of Tregs during *M. tuberculosis* infection [159, 160]. Despite the fact Tregs are believed to suppress protective immunity, a serious contraction of this population might negatively affect CD4 T-cell priming and activation [161]. Thus, although PD-1 signalling seems to be important in the TB immune response, the mechanisms involved may be complex and context specific.

### PD-1-expressing CD4 T-cells associate with protective responses

A growing body of data suggests IC expression, including PD-1, may not simply be a marker of exhaustion, but instead be necessary to maintain T-cell function in TB. A landmark study by Sakai *et al.* [162] showed twice as many *M. tuberculosis*-specific CD4 T-cells in the lungs of mice are retained in the vasculature compared with the parenchyma. Those CD4 T-cells in the parenchyma exhibited higher levels of PD-1 expression, whereas those in the vasculature predominately expressed killer cell lectin like receptor G1 (KLRG1), a marker of terminal differentiation [163]. The vasculature subset expressed higher levels of T-bet and produced IFN-γ more robustly. However, when transferred into *M. tuberculosis*-infected T-cell-deficient mice the parenchymal PD-1^hi^KLRG1^lo^ CD4 T-cells migrated back into the parenchyma where they restricted bacterial growth more effectively than the PD-1^lo^KLRG1^hi^ vascular counterpart. Likewise, vaccination with the H56 subunit conferred superior protection against subsequent *M. tuberculosis* challenge, linked to the induction of PD-1^+^KLRG1^−^ CD4 T-cells [164]. This PD-1^+^KLRG1^−^ subset was highly proliferative, homed to the lung, and produced more IL-2 and IL-17 and less IFN-γ and TNF-α than PD-1^−^KLGR1^+^ subsets, which had a shorter lifespan and could not proliferate upon adoptive transfer into *M. tuberculosis*-infected host [165]. Consequently, *M. tuberculosis*-specific PD-1^+^ CD4 T-cells confer greater protection than KLRG1^+^ counterparts when adoptively transferred [166]. In a separate study examining the importance of antigen specificity, multiple infusions of the *M. tuberculosis* antigens early secreted antigenic target 6 kDa (ESAT-6) or Ag85B led to an upregulation of PD-1 on CD4 T-cells specific for both antigens, but only Ag85B led to a reduction in bacterial burden in the lung [167]. Together these data, showing that functional and protective T-cells can express PD-1, indicate that it is not strictly a marker of exhaustion on TB-specific T-cells, although the mechanistic details are not clear. In contrast, exhausted CD8 T-cells expressing high levels of PD-1, TIM-3 and LAG-3 produced less IFN-γ and IL-2, and correlated with impaired control of *M. tuberculosis* replication [168]. The presence of CD4 T-cells was necessary to prevent exhaustion and promote effector CD8 T-cells, resulting in enhanced recognition of *M. tuberculosis*-infected macrophages. Detailed investigation in macaques revealed *M. tuberculosis*-specific CD4 T-cells expressed higher levels of PD-1 and CTLA-4 in the granuloma but failed to penetrate within the bacilli-containing core [169]. Recently, the same group found that PD-1 blockade did not affect CD4 T-cell penetration into the centre of granulomas, which suggests that PD-1 expression does not impair T-cells trafficking into this region [150]. These findings stress the importance of the positioning of the protective response at the site of infection and may mirror the lack of PD-1 expression around necrotic granuloma observed in humans.

## Implications of ICI in TB

The observation that ICI re-activates TB raises interesting questions about disease pathophysiology and some of the basic assumptions made with regard to protective immunity. Most importantly: why is ICI effective as a cancer therapy yet leads to re-activation of TB? It is plausible that pathogen-specific characteristics allow *M. tuberculosis* to thrive in a hyperinflammatory environment [77], such as promoted by ICI. Lung destruction is a well-characterised hallmark of pulmonary TB in active pulmonary TB and is mediated by matrix metalloproteinases (MMPs) [170, 171]. Cavitation is thought to be a key process in generating bacilli-containing aerosols and facilitating transmission, and *M. tuberculosis* may be under selective pressure to maintain a vigorous T-cell response to drive this process [172, 173]. Therefore, increased Th1 responses brought on by ICI could exaggerate cytokine release and MMP expression, leading to tissue degradation and upsetting the delicate balance of host–pathogen interactions that exist in the lung. This concept is supported by observations that different inhibitory receptors also act as vital modulators of TB immune control. Mice lacking the chemokine scavenger D6 or the negative regulator of the IL-1 system, Toll/IL-1 receptor 8 (TIR8), were rapidly killed by low-dose *M. tuberculosis* challenges accompanied by considerable local and systemic inflammation [174, 175]. Interestingly, both studies showed no differences in *M. tuberculosis* growth kinetics between KO and wild-type groups, suggesting that TB disease was driven by hyperinflammation not bacterial growth. In humans, classical epidemiological surveys showed that a strong tuberculin response in childhood is associated with an increased risk for TB disease later in life, implying disease associates with heightened immune responses [176]. Therefore, the restoration of effector functions observed with ICI *in vitro* may exacerbate disease rather than improve control (figure 2). In addition, suppression of PD-1 and CTLA-4 signalling has been linked to autoimmunity as a result of aberrant T-cell activation [3]. Autoreactive T-cells are reported to be increased in TB patients and the granulomatous pathology is often indistinguishable from that observed in the autoimmune disease sarcoidosis [177, 178]. Plausibly, ICI might tip the balance further towards autoreactivity and increased tissue destruction, although the fact that CTLA-4 blockade does not seem to cause this may argue against this hypothesis.

On the other hand, some of the data presented earlier, particularly from animal models, imply that PD-1 expression may actually correlate with enhanced resistance to *M. tuberculosis*. Conceivably, TB latency is maintained by PD-1^+^ T-cells that are functionally restrained. In this scenario, PD-1 blockade leading to T-cell dysfunction characterised by hyperinflammatory responses resulting in breakdown of immune control. One important point to highlight in this regard is that of timing. The PD-1 experiments in TB animal models highlighted here are probably more representative of primary human TB infection [179], while ICI-associated TB in humans is almost certainly post-primary disease. PD-1-expressing T-cells may have different roles and functional capacities in these different disease states, as may other immune subsets expressing ICIs. The fact anti-PD-1 worsens disease when administered early during infection in mice and monkeys, as well as in latent humans, indicates that PD-1 is critical to regulation of immunity at both stages of infection. Although the mechanistic details are not clear and the data can be contradictory, several independent studies suggest that PD-1 expression is linked to T-cell homing into the lung parenchyma, *i.e.* the site of infection. Moreover, in these studies, protective PD-1^+^ T-cells which were positioned in the parenchyma produced less IFN-γ than their PD-1^−^ counterparts; thus, along with the quality of immune responses, these findings suggest the importance of their spatial organisation. Therefore, although ICI *in vitro* appears to restore classic *M. tuberculosis*-specific Th1 functions (figure 2), these functions may not be the suitable ones, at least in certain contexts. In addition, PD-1 blockade may negatively impact some aspects of protective immunity.

Finally, it is important to note that TB re-activation is a side-effect of ICI for cancer and, to the best of our knowledge, ICI has never been used primarily as TB therapy in humans. Potentially, therefore, the dosages used in cancer therapy are inappropriate for enhancing TB immunity and adjusting the dosages for TB treatment could produce a beneficial effect. Keeping in mind that ICs act more as a dial instead of a switch, the effects on *M. tuberculosis* infection might vary at different concentrations of inhibitors [66, 67]. In addition, re-activation induced by PD-1 blockade occurred in the absence of antibiotics. It is possible that PD-1 blockade or other ICI could be leveraged together with conventional TB drug therapy to bolster sterilisation of latent or indeed active TB infection. More inhibitors are currently under investigation for use in cancer therapy and their usefulness in TB remains to be explored [106]. Of the potential candidates, experimental data suggest that TIM-3-blocking agents in particular might have some benefits for use in TB. Contrary to PD-1 KO mice, TIM-3 deficiency increased survival upon TB infection and inhibition of this pathway reduced bacterial burden [143]. Experimental data is lacking for this and many of the other IC pathways, and further investigation is required to determine the potential for targeted ICI to improve host control of TB.

## Conclusions

The current paradigm that driving increased Th1 responses may lead to greater control of TB is challenged by the emerging evidence on anti-PD-1-associated TB re-activation (figure 3). Overall, in view of the observations of TB re-activation after anti-TNF-α therapy, on the one hand, and anti-PD-1 therapy, on the other, it appears that a window of protective immunity exists in humans, sitting in between two extremes of insufficient and excessive T-cell immunity [180]. This optimally balanced response represents effective immune control which robustly contains *M. tuberculosis* circumscribed by vital negative regulation to prevent the scales tipping in favour of hyperinflammation and disease progression. Whether ICI can be leveraged to fine-tune this response remains to be seen.

## Shareable PDF

10.1183/13993003.02512-2021.Shareable1 This one-page PDF can be shared freely online.
Shareable PDF ERJ-02512-2021.Shareable
