# Supplementary material for: The paradox of immune checkpoint inhibition re-activating tuberculosis
Source: Eur Respir J. 2022 Nov 10;60(5):2102512. doi: 10.1183/13993003.02512-2021 (PMC9647068; doi:10.1183/13993003.02512-2021)
Supplement: Supplementary file 1 [file ERJ-02512-2021.Shareable.pdf]

SHAREABLE PDF

The paradox of immune checkpoint inhibition re-activating tuberculosis

Mohamed Ahmed[1,2], Liku B. Tezera[3,4], Paul T. Elkington[3,5] and Alasdair J. Leslie[1,2,4]

[1]Africa Health Research Institute, Durban, South Africa. [2]College of Health Sciences, School of Laboratory Medicine and Medical Sciences, University of KwaZulu-Natal, Durban, South Africa. [3]NIHR Biomedical Research Centre, School of Clinical and Experimental Sciences, Faculty of Medicine, University of Southampton, Southampton, UK. [4]Dept of Infection and Immunity, University College London, London, UK. [5]Institute for Life Sciences, University of Southampton, Southampton, UK.

Corresponding author: Alasdair J. Leslie (al.leslie@ahri.org)

Check for updates

Shareable abstract (@ERSpublications)
Immune checkpoint inhibition is employed as a host-directed therapy for cancer, but many reports have shown it can cause re-activation of latent TB. This observation warrants a reappraisal of protective TB immunity and drivers of re-activation. https://bit.ly/3vi2xu0

Cite this article as: Ahmed M, Tezera LB, Elkington PT, *et al*. The paradox of immune checkpoint inhibition re-activating tuberculosis. *Eur Respir J* 2022; 60: 2102512 [DOI: 10.1183/13993003.02512-2021].

This single-page version can be shared freely online.

Copyright ©The authors 2022.

This version is distributed under the terms of the Creative Commons Attribution Licence 4.0.

Received: 17 Sept 2021
Accepted: 6 April 2022

Abstract

By attenuating T-cell activation, immune checkpoints (ICs) limit optimal anti-tumour responses and IC inhibition (ICI) has emerged as a new therapy for a broad range of cancers. T-cell responses are indispensable to tuberculosis (TB) immunity in humans. However, boosting T-cell immunity in cancer patients by blocking the programmed cell death 1/programmed cell death ligand 1 (PD-1/PD-L1) axis can trigger re-activation of latent TB. This phenomenon appears to contradict the prevailing thought that enhancing T-cell immunity to *Mycobacterium tuberculosis* will improve immune control of this pathogen. In support of this anecdotal human data, several murine studies have shown that PD-1 deficiency leads to severe TB disease and rapid death. These observations warrant a serious reconsideration of what constitutes effective TB immunity and how ICs contribute to it. Through restraining T-cell responses, ICs are critical to preventing excessive tissue damage and maintaining a range of effector functions. Bolstering this notion, inhibitory receptors limit pathology in respiratory infections such as influenza, where loss of negative immune regulation resulted in progressive immunopathology. In this review, we analyse the mechanisms of ICs in general and their role in TB in particular. We conclude with a reflection on the emerging paradigm and avenues for future research.
